# The dynamic nature of electrostatic disorder in organic mixed ionic and electronic conductors†

**DOI:** 10.1039/d4mh00706a

**Published:** 2024-08-07

**Authors:** Colm Burke, Alessandro Landi, Alessandro Troisi

**Affiliations:** a Department of Chemistry, University of Liverpool Liverpool L69 3BX UK atroisi@liverpool.ac.uk; b Dipartimento di Chimica e Biologia “Adolfo Zambelli”, Università di Salerno, Via Giovanni Paolo II I-84084 Fisciano Salerno Italy

## Abstract

Charge dynamics in disordered media is described invariably assuming that the energy landscape for hopping site energy is stationary. Within the same framework, the correlation between low electronic disorder and high charge mobility is considered extremely robust, despite the emergence of materials with mixed ionic and electronic conductivity (OMIECs) that display high mobility coexisting with large disorder. We show in this work that the disorder of OMIEC polymers is highly dynamical, *i.e.* the on-site energy for charge transport fluctuates with a characteristic time comparable with that of electron transport. Under these conditions, the disorder of the “frozen” system is not relevant for the charge carrier, whose dynamics are instead controlled by the underlying dynamics of the material. Deep traps exist but have a finite lifetime. The combination of classical simulations and quantum chemical calculations on the nanosecond timescale seems ideal to disclose and characterise the phenomenon.

## Abstract 2

The disorder in organic mixed electronic and ionic conductors (OMIECs) is highly dynamic, and, consequently, charge transport is not adversely affected by it. The dynamics of the soft materials drives the charge carriers.

## Graphical abstract

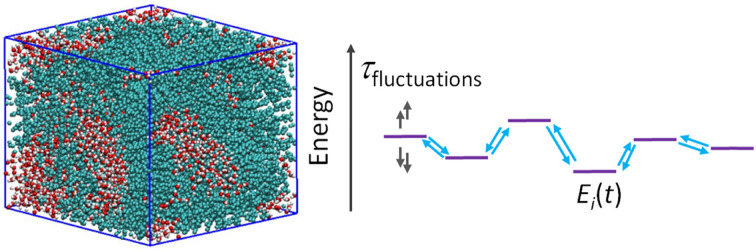

**New concepts**

It is generally believed that disorder in polymeric materials governs the dynamics of electronic charge. For this reason, it has been challenging to explain the observed high charge carrier mobility in organic mixed electronic and ionic conductors (OMIECs), which display good mobility and, at the same time, considerable conformational and electrostatic disorder. Our study solves this apparent contradiction by revealing that the disorder in these materials is highly dynamic, and, consequently, charge transport is not adversely affected by it. This observation changes the way we describe charge transport in OMIECs. Their energy levels fluctuate dynamically in time due to the structural flexibility of the material and the motion of surrounding water and ions. Such dynamics allows for frequent favourable alignment of the energy levels that enables the charge carriers to move much more rapidly because, in effect, electronic traps in these materials are short-lived.

The resurgence of interest in organic mixed ionic and electronic conductor (OMIEC) materials has naturally led to a growing body of work employing simulation methods. These studies are varied in scope, but most have a general focus on characterising the microstructure/morphology of these materials through classical simulations (*e.g.*, swelling studies,^1,2^ side-chain engineering,^3,4^ ion coordination^5^), such that few have combined molecular dynamics (MD) with electronic structure calculation^6^ and, to the best of our knowledge, none have computed the density of states (DOS) for atomistic models of such systems. In conventional, dry organic semiconductors, it can be assumed that the DOS is static both in time and with the level of doping.^7,8^ In OMIECs, this is not necessarily the case, due to (i) the presence of an electrolyte, (ii) the fact that ions and water are dispersed in the polymer phase,^9^ and (iii) intrinsic differences in chemistry in comparison to conventional semiconductors. The current lack of a characterisation of the “bulk” electronic structure (as an ensemble of conformations from MD) means that the effects of these factors on the electronic disorder and hence transport properties of these materials have not been elucidated, precluding the development of a theory of charge transport. To address this issue, we utilise in this paper quantum chemical (QC) calculations performed on MD trajectories in an effort to model the static and dynamic disorder of a typical mixed-conducting polymer, with the aim of quantifying the extent to which the dynamics of water and ions influence the shape of and the fluctuations in the DOS, and hence the movement of electrons.

The system chosen for this task is an oligomer model of poly(2,5-bis(3-triethylene-glycoloxythiophen-2-yl)-*co*-thiophene), p(g2T-T), the repeat unit of which is shown in Fig. 1a. This polymer is a benchmark^10^ in – and representative of – the conjugated polymer electrolyte class of OMIECs, which have advantages in their accumulation mode operation^11^ and lack of phase separation^12^ when compared with polymer/polyelectrolyte blends. An initial system of 64 p(g2T-T) chains, 2760 water molecules (30% by weight) was generated, with 31 pairs of sodium and chloride ions (approx. 0.5 M) randomly inserted into the simulation box. The force-field implemented is identical to that used in ref. 6, and the reader is referred there for further details. A certain number of chains were then charged (or neutralised) according to the desired doping level of the system and the corresponding number of chloride ions was randomly inserted (or removed) in each case to maintain neutrality. In the case of the bulk system, all water molecules were also removed. Sub-T_G_ annealing simulations^13^ were used to accelerate the equilibration of each system (see Fig. 1b and Section S4, ESI† for details and further verification), with the average end-to-end distance of polymer chains used as a measure of progress towards equilibrium (Fig. 1c) – as has been done in previous studies.^14^ A visualisation of the equilibrated neutral system is depicted in Fig. 1d, whose inspection clearly shows the partitioning of electrolyte and polymer into two distinct phases. Fig. 1e depicts a schematic of the energy levels in a disordered semiconductor, whose distribution and dynamics are investigated in this work.

**Fig. 1:**
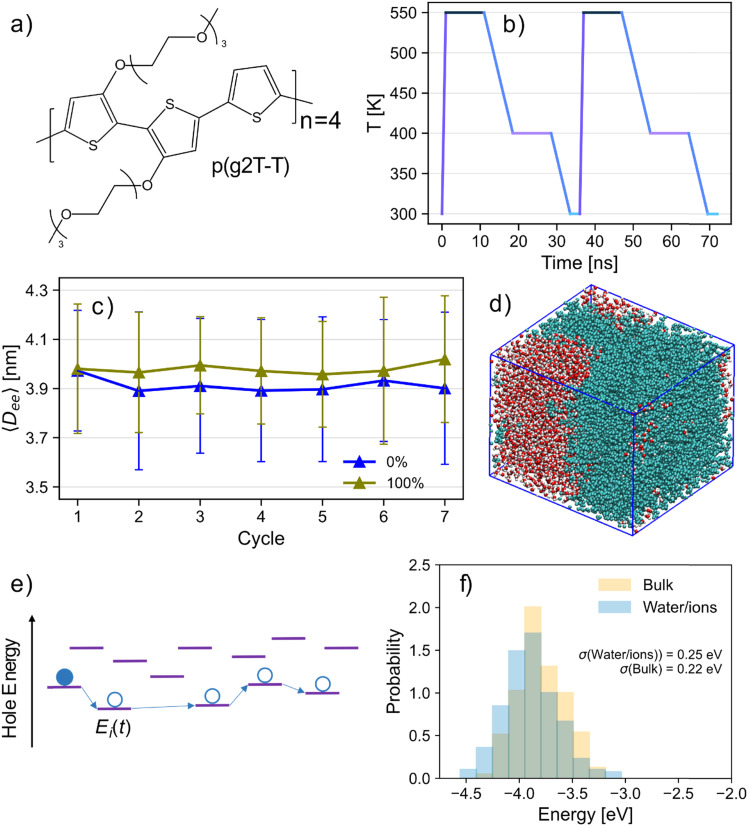
(a) Chemical structure of the repeat unit of p(g2T-T) (b) schematic of two annealing cycles. (c) Mean and standard deviation of polymer chain end-to-end distance at 300 K after each of seven annealing cycles of neutral (blue) and fully charged (olive) systems. (d) Visualisation of the neutral simulation box. The separation between the polymer phase (light blue) and the electrolyte phase (red and white) is clearly visible. (e) Schematic of the energy levels in a disordered material and the expected path followed by a charge carrier. The on-site energies are normally considered static - in this paper we evaluate their distribution and dynamics. (f) Comparison of the HOMO energy distribution of bulk-phase (yellow) p(g2T-T) with the distribution in the presence of water and ions (light blue) for 250 samples.

QC calculations were performed in open boundary conditions by translating the (neutral) polymer chain of interest to the centre of the simulation box, with all surrounding atoms included as electrostatic point charges. In a preliminary set of calculations, the ionisation potential (IP) of the chain is computed as the (vertical) energy difference between the energies of the chain with net charge +1 and the neutral chain. As shown in the ESI,† Section S1, the same information can be obtained with very good accuracy by using Koopmans’ theorem and simply computing the HOMO of the neutral chain. This computationally cheaper approach was followed to obtain the data reported in the main manuscript. We reported the results obtained at the B3LYP/3-21g* level and verified in the ESI† that the results correlate extremely well with those of other functionals (Fig. S2, ESI†). The IP distribution of all chains indicates which chain is easiest to dope and provides an energy landscape for charge migration from chain to chain. To investigate the effect of polarisation on the obtained results for the systems with net charge, we adapt a combined QM/MM+MD method introduced in ref. 6 to model the reorganisation of excess charge on charged chains in response to changes in the environment. Since the simulation timescales achievable by this protocol are limited, and longer timescales are needed to allow comparison with results derived from purely classical MD trajectories, in the QC part the DFT calculations used in ref. 6 were replaced in this work with the self-consistent charge density functional tight-binding method (SCC-DFTB).^15^ SCC-DFTB has been used similarly in the past to model the coupling between a quantum mechanically embedded region and a classically propagated aqueous environment – notably for biomolecular systems^16,17^ and, more recently, in graphene-electrolyte simulations.^18,19^ In our case, this approach achieved a remarkable increase in simulation speed (10 ns day^−1^*vs.* 0.5 ns day^−1^ using the former method) while sacrificing very little in terms of accuracy (see the ESI,† Section S3, for validation of this method and further details).

Fig. 1f shows the distribution of chain HOMO energy for bulk and aqueous neutral systems. The distribution is somewhat broadened in the presence of electrolyte and, despite the average HOMO energy being more negative in this case (which corresponds to increased IP), the polymer in electrolyte exhibits an increased likelihood of occupying states at both low- and high-energy extremes relative to the bulk system. The bulk results for p(g2T-T) are reported in this work to highlight the much broader distribution compared to those calculated for bulk models of conventional semiconducting polymers (SCPs) (taken from previous work in ref. 20). The standard deviation of HOMO energy (indicated with *σ* in Fig. 1f) for equivalent sampling of the polymer DPP-BTz (0.14 eV) is roughly half of that of p(g2T-T), and IDT-BT shows even smaller energetic disorder (0.09 eV). The results of the bulk rule out the possibility that the large broadening is due to water and electrolyte. A possible explanation was that the greater electronic disorder is due to a larger intrinsic disorder due to the conformation of p(g2T-T) but calculation of σ for the backbone in the absence of the electrostatic effect of the surrounding show that this is very small (0.09 eV shown in Fig. S5 of the ESI†). We conclude from these analyses that the broadening is due to the interaction of the backbone with the oligo(ethylene glycol) (OEG) side-chains – present only in p(g2T-T).

To investigate the dynamics of the electronic disorder of the neutral system in electrolyte, the fluctuation of chain HOMO energy was computed in 5 ps intervals over a total simulation time of 2.5 ns. Fig. 2a shows the HOMO energy distribution over the course of the simulation, with the Fig. 2b showing its variation for one chain in each quartile of the initial distribution over the first 400 ps. As can be seen, within the length of the simulation each chain spans a substantial fraction of the HOMO energy distribution, but it is also clear that chains retain some “memory” of their initial conformation and environment on this timescale. The reason for this is because, while the short-term electrostatic environment is highly dynamic due to the movement of water/ions and side-chains around the backbone, on the nanosecond timescale the HOMO energy shows no appreciable variation due to the limited movement of the backbone itself at 300 K. To quantify the multiple timescales involved we report in Fig. 3b the autocorrelation function (ACF) of chain HOMO energy, defined here for time lag *k* as *Ẽ*_*k*_ = (*E*_*t*_ − *Ē*)(*E*_*t*−*k*_ − *Ē*), where *Ē* is the average HOMO energy across all chains and all time lags and *Ẽ*_*k*_ is averaged first over all values of *t* and then over all chains considered. The ACF decays almost immediately (10 ps) to ∼50% of its initial value, but begins to plateau at 200–400 ps over the timescale shown. In essence, we distinguish (i) fast fluctuations due to local displacement of atoms (also noted in ref. 21), (ii) intermediate fluctuations in the timescale of diffusion of water and ions, and (iii) conformational changes that are slow enough so as to give the appearance of a disorder that is static in the timescale of the simulation.

**Fig. 2:**
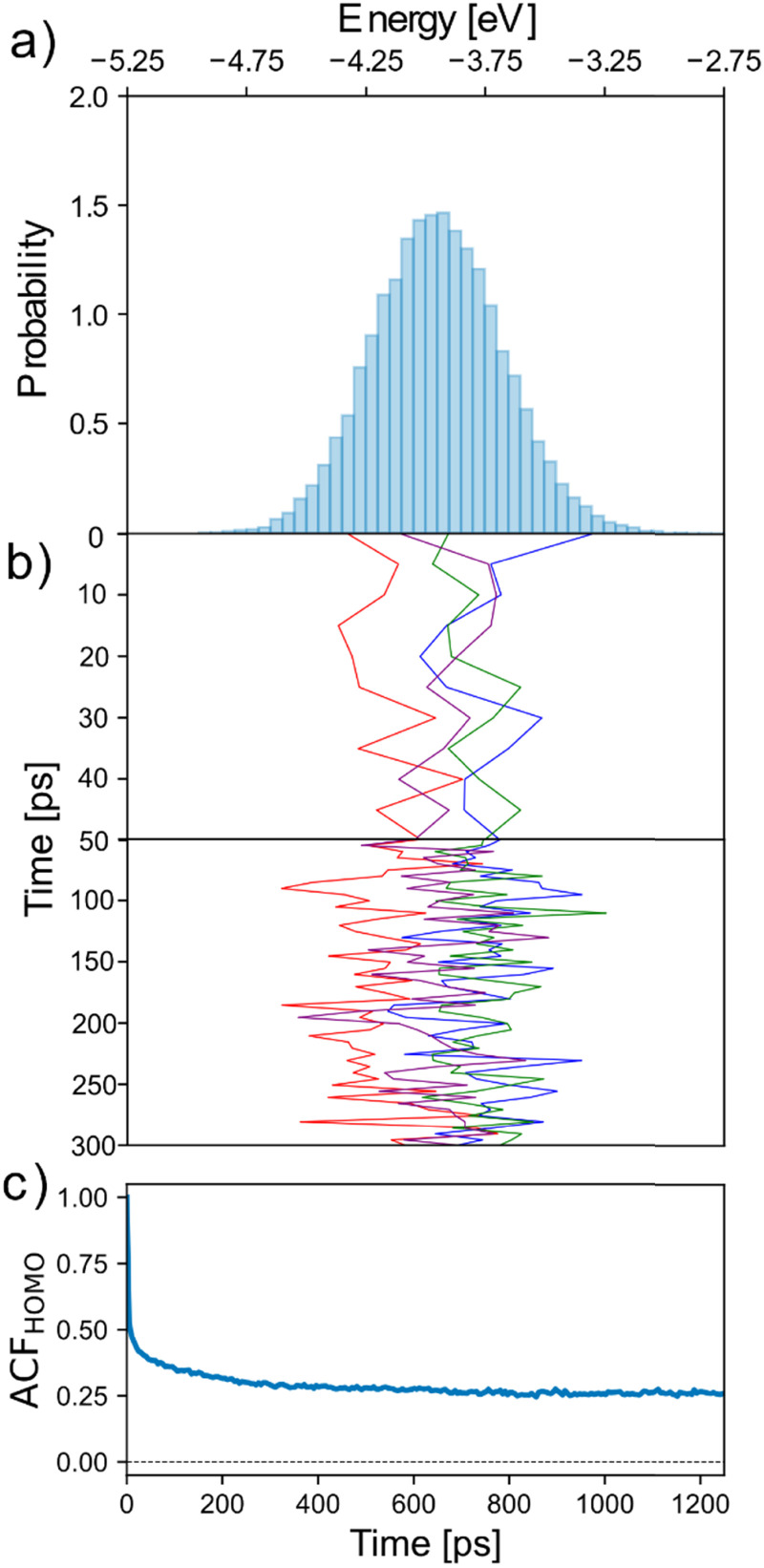
(a) HOMO energy distribution across 2.5 ns simulation time, normalised such that the total histogram area is equal to one. (b) Fluctuation in HOMO energy for four chains spanning the range of the initial energy distribution in panel (a) across the first 300 ps of the simulation. (c) Autocorrelation function of chain HOMO energy using half the simulation length as the largest chosen time lag, and the same with 300 ps as the largest chosen time lag (inset).

**Fig. 3:**
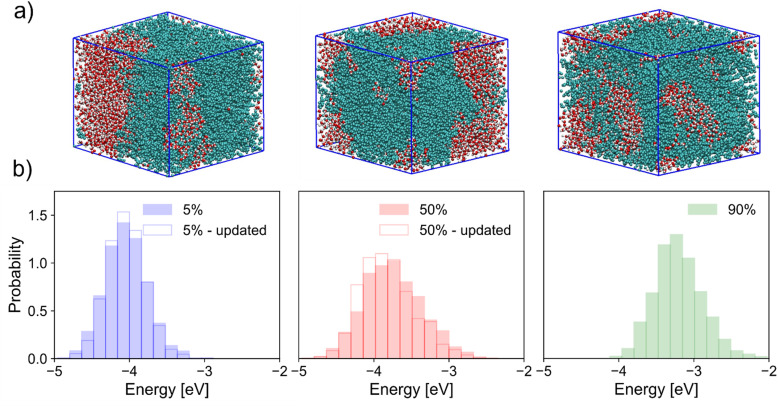
(a) Visualisation of simulation box for systems with 5% (left), 50% (middle), and 90% (right) of chains charged. Polymer chains are coloured light blue and clusters of water in red and white. (b) Corresponding neutral chain HOMO energy distributions for each doped system. Distributions with excess charge distribution updated in response to the local environment are also shown for lower doping as transparent histograms with bold edges while those with static excess charge are filled.

Fig. 3a shows visualisations of the simulation box for three levels of doping of polymer chains. As has been demonstrated many times before,^22,23^ doping in mixed-conducting materials is concomitant with inherent changes in material microstructure; this is reflected here in the clear mixing of polymer and water phases due to vehicular transport of chloride ions and water – facilitated by OEG side-chains.^24,25^ Because of these changes in morphology, the DOS of neutral chains is modified as the doping level increases, as shown in Fig. 3b. These results are reported both for trajectories with a static distribution of the excess charge on charged chains, and for trajectories where the excess charge has been allowed to rearrange in response to changes in the environment, according to the method explained earlier. While having little impact on the conclusions drawn here, inclusion of polarisation effects nevertheless noticeably changes the DOS of doped systems. In general, this is manifest in a narrowing of the HOMO energy distribution, and an (on average) stabilisation of the neutral state in comparison to a static characterisation. These effects are fairly small when looking at calculations on a large number of chain conformations, but are likely to have a larger impact on mechanistic studies and on those with a stronger focus on material microstructure. In Fig. 3b we note a possibly counterintuitive shift of HOMO levels to higher energy (a stabilisation of the cationic state) as charge density increases. This result can be rationalised (as shown in the ESI,† Section S6) by considering the increased variance in electrostatic potential (EP) along a chain with increased surrounding charge density. The hole in the cationic state localises preferentially on a site where it is most stable, and this stabilisation is on average greater when the fluctuation in EP is greater, *i.e.* when the doping level is larger. Another observation that can have an impact on the modelling of the charge dynamics is that the extent to which the DOS is broadened is largest at an intermediate doping level, which can be attributed to a maximum in the heterogeneity of the electrostatic disorder. At large doping levels screening effects become important, *i.e.* there is a maximum effect of the additional charges on the broadening and we should remember that a dominant component of the broadening is due to the glycol side chains. The effect of doping on the dynamics of the energy levels of individual chains (rather than the global DOS) follows a slightly different trend. To compare the extent of this dynamic disorder at each level of doping considered, the mean standard deviation of HOMO energy for each neutral chain was calculated for successive 250 ps slices of trajectories analogous to the one considered for the neutral system in Fig. 2, which yields values of 0.24 eV and 0.29 eV for systems with 50% and ∼90% of chains in their cationic state, respectively, compared to 0.20 eV for the neutral system. In other words, the dynamic disorder increases monotonically with doping.

The consequences of the dynamic nature of the on-site energy revealed by the computations are dramatic. A so far universal assumption in the description of charge dynamics in disordered media is that the landscape of hopping sites is stationary,^26,27^ except for the possible role of charge–charge interactions.^28–30^ We have observed instead that the amplitude of the fluctuations of the on-site energy is very large and comparable with the overall magnitude of on-site disorder, and the timescale of such fluctuations is comparable or faster than the expected hopping rate (tens of ps). The influence of conformational changes on the rate is well known in biophysics: the effect of dynamically changing on-site energy is that the hopping rate cannot be described by a simple rate equation^31,32^ and the observed dynamics reflects the dynamics of conformation change rather than the rate of electron transport. Dynamic change of the on-site energy can wash out the effect of static disorder as the carrier can “wait” for a favourable energy level alignment before hopping and the dynamics is therefore determined by the waiting time rather than the intrinsic hopping rate.

To illustrate this more quantitatively, we developed a toy model made by a 1D array of sites where charge is allowed to hop to its nearest neighbour with a rate depending on the energy difference between the sites (a standard Miller–Abrahams dependence – see Fig. 4a), and on the characteristic hopping time *τ*_0_, conveniently set to be the unit of time in the model. The on-site energies are distributed normally with standard deviation *k*_B_*Tσ*_E_ and evolve in time stochastically (as an overdamped Langevin oscillator)^33^ with characteristic fluctuation time *τ*_fluct_ to reproduce the computational observation. The charge is placed initially on a site with probability given by the Boltzmann distribution, *i.e.* it is typically “trapped” in the low energy tail of the DOS. We monitor the escape time *τ*_escape_, defined as the time required for the population to become 50% of the original population, as a function of the disorder *σ*_E_ and the fluctuation time *τ*_fluct_ (details on the numerical implementation of the model are given in the ESI,† Section S7). As shown in Fig. 4b, for fast fluctuations, the escape time becomes independent from the disorder of the system because it is faster for the charge to wait until the energy levels align favourably and the disorder becomes irrelevant. For intermediate fluctuation times, a dependence on the disorder *σ*_E_ develops but this is much weaker than the exponential dependence expected when there is no on-site energy fluctuation.^7,34^ According to this model, fluctuations with characteristic time up to 3 orders of magnitude slower than the faster hopping time *τ*_0_ must be included in the modelling if one is trying to establish a correlation between microscopic structure and transport.

**Fig. 4:**
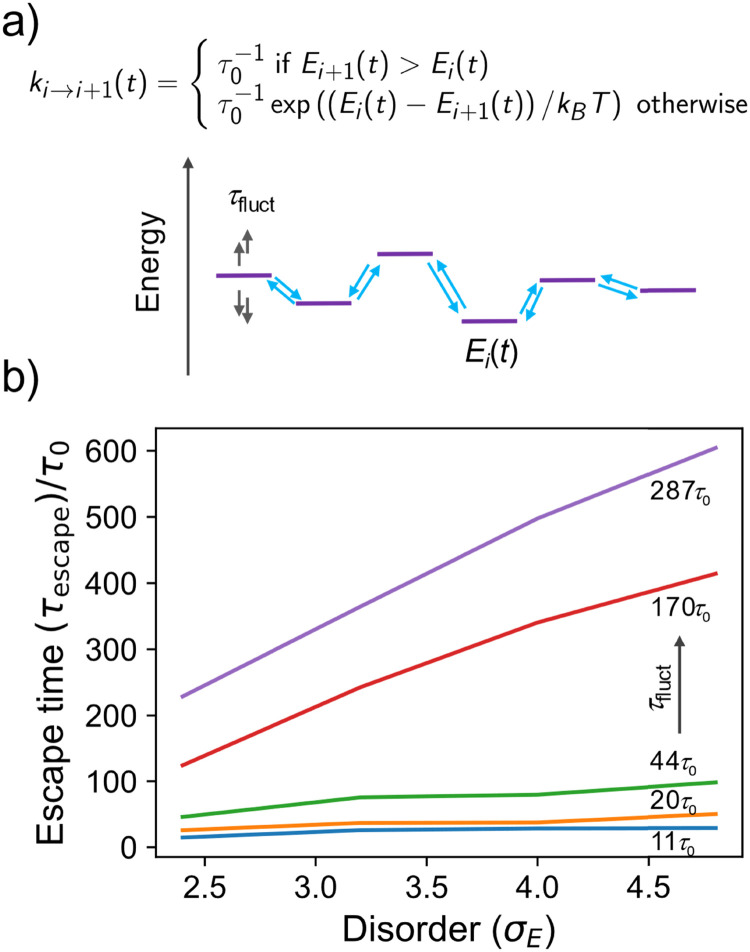
(a) Schematic of a 1D kinetic model with nearest neighbour hopping where the on-site energy fluctuates with characteristic time *τ*_fluct_ and the characteristic hopping time is *τ*_0_. (b) Escape time (defined in text) of a charge carrier as a function of the disorder *σ*_E_ for different values of fluctuation time *τ*_fluct_= 11, 20, 44, 170 and 287 *τ*_0_.

The picture emerging from this work allows us to rationalise the relatively high charge mobilities displayed by the best OMIEC materials^10^ (in the region of 0.9 cm^2^ V^−1^ s^−1^), which have been considered at odds with the large conformational and electrostatic disorder expected for such systems. This paper, while confirming the larger magnitude of electrostatic disorder in OMIEC, has shown that the disorder is highly dynamical and, as a consequence, the transport is not negatively impacted by it. The coupling between nuclear (ionic) and electronic degrees of freedom is not only a feature of the devices but a fundamental aspect of the transport mechanism in these materials. For the same reason, molecular dynamics simulations, mainly used to produce plausible microscopic models for organic electronics, are expected to become prominent players in the characterization of OMIEC because the nuclear dynamics at the atomic scale is precisely what determines the electron dynamics.

## Data availability

The data supporting this article have been included as part of the ESI.†

## Conflicts of interest

There are no conflicts to declare.

## Supplementary Material

MH-011-D4MH00706A-s001
