# Supplementary material for: The dynamic nature of electrostatic disorder in organic mixed ionic and electronic conductors
Source: Mater Horiz. 2024 Aug 7;11(21):5313–9. doi: 10.1039/d4mh00706a (PMC11320175; doi:10.1039/d4mh00706a)
Supplement: MH-011-D4MH00706A-s001 [file MH-011-D4MH00706A-s001.pdf]

Supplementary Information (SI) for Materials Horizons.
This journal is © The Royal Society of Chemistry 2024

Supporting information for:

The dynamic nature of electrostatic disorder in organic mixed ionic and electronic conductors

Colm Burke[1], Alessandro Landi[1,2], and Alessandro Troisi[1*]

[1] Department of Chemistry, University of Liverpool, Liverpool, L69 3BX, UK.
[2] Dipartimento di Chimica e Biologia "Adolfo Zambelli", Università di Salerno, Via Giovanni Paolo II, I-84084 Fisciano, Salerno, Italy

*Corresponding Author:
Alessandro Troisi
Email: atroisi@liverpool.ac.uk

S1. Justification for use of HOMO energy in manuscript

Due to a 50% reduction in computational burden, in the main manuscript we use the HOMO energy of a polymer chain as an approximation of its ionisation potential, and thus as a measure of the ease of oxidation of p(g2T-T) chains. The strong correlation of ionisation potential with the absolute value of the HOMO energy of chains (shown in Figure S1) confirms that this approximation is reasonable, at least for chains of the length used in this study.

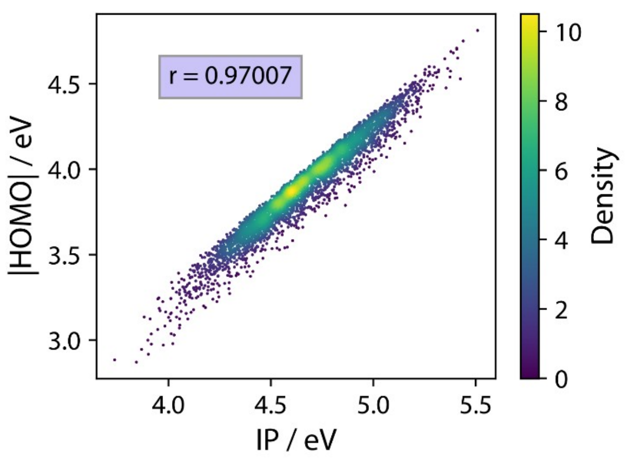

Figure S1: Correlation of the absolute value of chain HOMO energy with chain ionisation potential for ca. 4000 samples.

S2. Comparison of results obtained for different functionals and basis sets

To confirm that the conclusions observed in the main manuscript do not change with long-range corrective functionals and are basis set independent, HOMO energies were calculated for a small sample of 64 neutral chains extracted from a simulation trajectory with 50% charged chains using CAM-B3LYP/3-21G*, LC-wHPBE/3-21G*, and LC-wHPBE/6-31G* levels of theory (in addition to the original B3LYP/3-21G*). Figure S2a shows a very strong correlation between all levels of theory. The correlation between two functionals (one with long-range correction) is plotted in Figure S2b, which shows an expected shift in absolute HOMO energies, but this does not influence any of the results as this shift is maintained for different levels of doping (also shown).

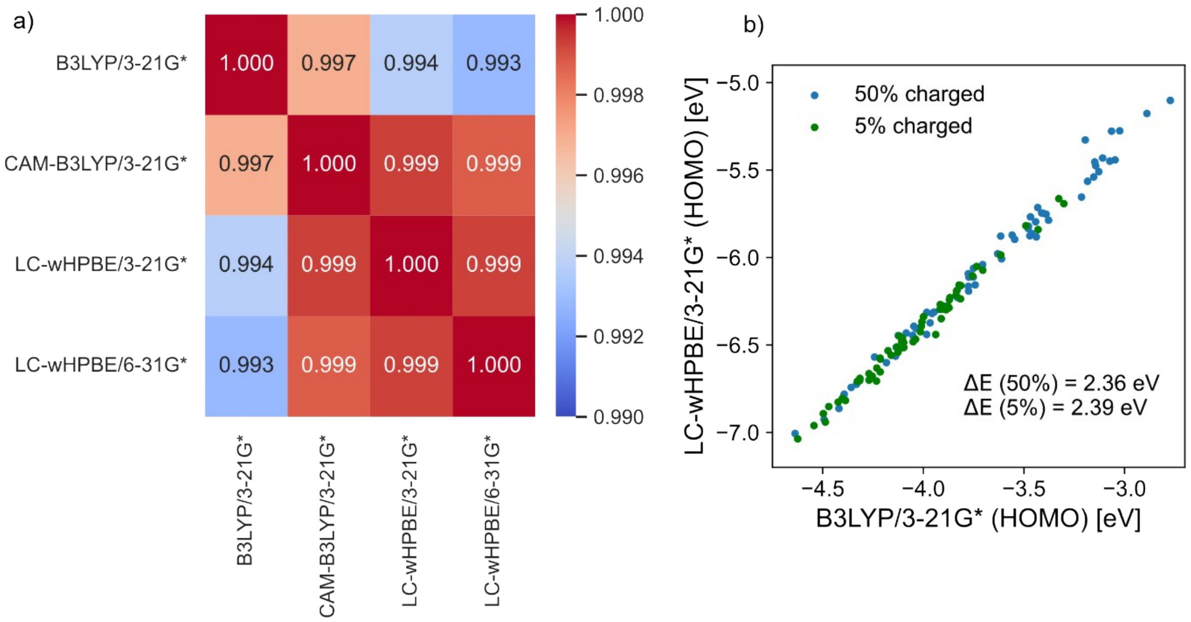

Figure S2: (a) Correlation heatmap for HOMO energy computed with different functionals and basis sets for a sample of 64 chains extracted from a simulation with 50% of the chains charged. (b) Scatter plot comparing the results of two functionals (one with long-range correction) sampling from 5% charged (green) and 50% charged (blue) simulations.

S3. Verification of DFTB implementation for charge-update

The original implementation[1] of the method used to update the excess charge along polymer chain backbones used the B3LYP/3-21G* level of theory for the QC part, achieving simulation speeds of approximately 0.5 ns^{-1} day^{-1} $node^{-1}$. Since in this study we need to generate trajectories much longer than those reachable in this implementation, the QC part of the scheme was replaced with the self-consistent charge density functional tight-binding method (SCC-DFTB), which increased performance to ~10 ns^{-1} day^{-1} $node^{-1}$. To verify that this method approximated – with sufficient accuracy – the results of the initial approach, the excess charge distribution was calculated for both levels of theory for 16 p(g2T-T) chains extracted from a single MD snapshot (see Figure S3). We note a close match in excess charge distribution in all but one case, where a minor difference is seen due to convergence to different states.

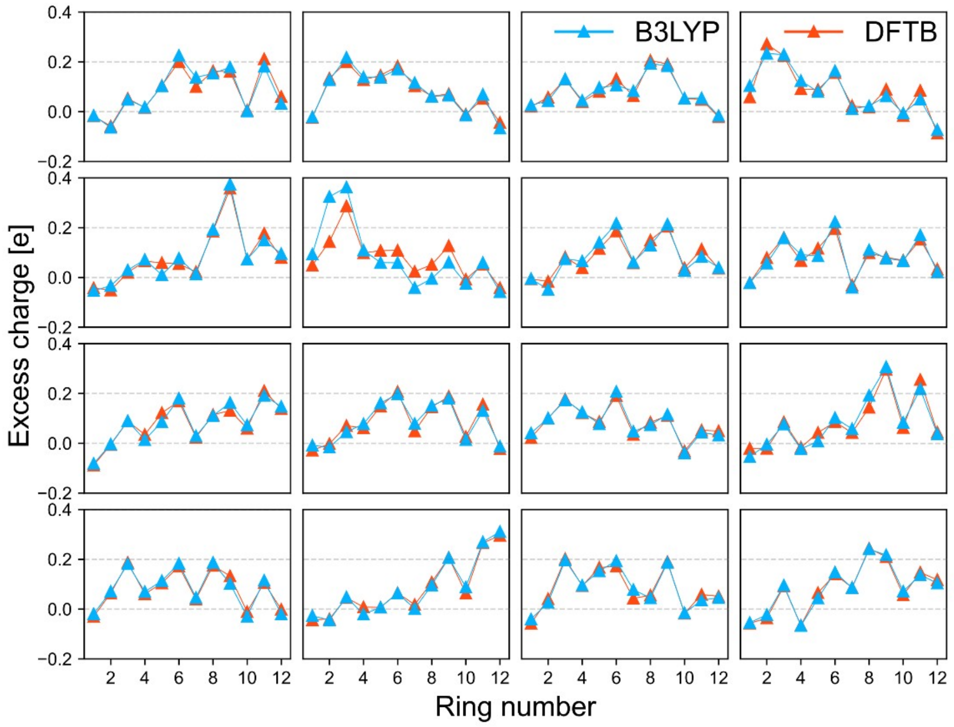

Figure S3: Computed excess charge over thiophenic rings for 16 cationic p(g2T-T) chains extracted from a single MD snapshot, using either B3LYP/3-21G* (blue) or SCC-DFTB (red).

S4. Additional equilibration figures

For the purpose of designing a suitable annealing protocol, the glass transition temperature T_G of p(g2T-T) was estimated via a bilinear fit of the specific volume-temperature curve (Figure S4a), using data derived from a simulation of the bulk polymer whereby the simulation temperature was gradually quenched (at 0.02 K/ps) from 900K to 100K. T_G was found to be 523K from this fitting, and thus the temperature of 550K used in the annealing protocol is reasonable. We also show the root mean square deviation of the centre of mass of p(g2T-T) chains at 550K in Figure S4b, which is ~1nm in 2ns and confirms that chains are no longer glassy at this temperature.

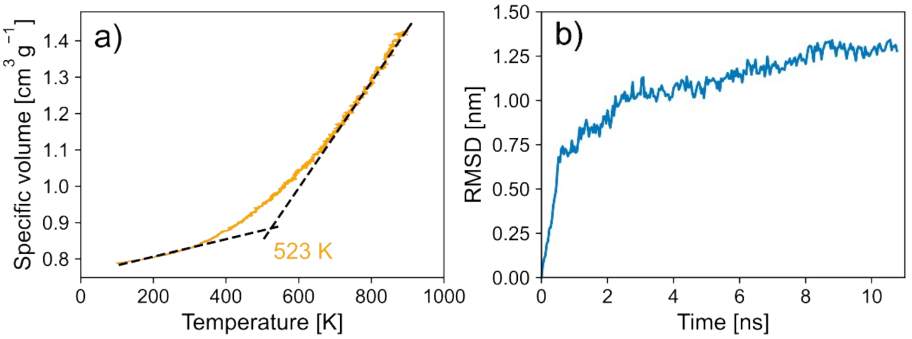

Figure S4: (a) Fits to the two linear regimes of the specific volume-temperature curve of bulk p(g2T-T). The glass transition temperature is estimated from the intersection between these fits. (b) Root mean square deviation of the centre of mass of p(g2T-T) at 550K.

S5. Additional calculations on ‘bulk’ system

Additional analysis was performed to further unravel the origin of the large electrostatic disorder observed in bulk p(g2T-T). To accomplish this, calculations were performed on chains extracted from

MD without inclusion of point charges (i.e., in vacuum) – essentially modelling only the disorder induced by the backbone conformation. In Figure S5 we plot the distribution of HOMO energy in vacuum alongside the distribution when all surrounding point charges are included (electrostatic embedding). The standard deviation σ is very small in comparison, which indicates that only a small proportion of the overall disorder derives from backbone flexibility, and the majority is due to interaction between the chain and oligo(ethylene glycol) (OEG) side-chains.

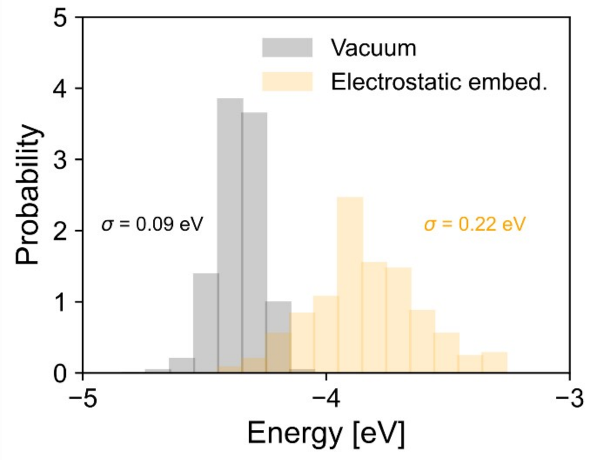

Figure S5: HOMO energy distributions for chains extracted from simulation of bulk p(g2T-T). 'Vacuum' refers to calculations performed on isolated chains and 'Electrostatic embed.' to those with all surroundings included as point charges.

S6. Model calculation for charged system results

To validate the positive shift of HOMO energies observed for the doped systems in Figure 3b of the manuscript, calculations were performed for a model p(g2T-T) chain whereby 100 samples were generated by placing two equal and opposite point charges at a random distance 4-10 Å away from the closest atom in the chain. The distribution of calculated HOMO energies for the 100 samples is shown in Figure S6a alongside the HOMO energy of the isolated chain. The mean of this distribution is shifted towards more negative values by 0.24 eV compared to the isolated chain, which demonstrates the – on average – stabilising impact of greater electrostatic disorder.

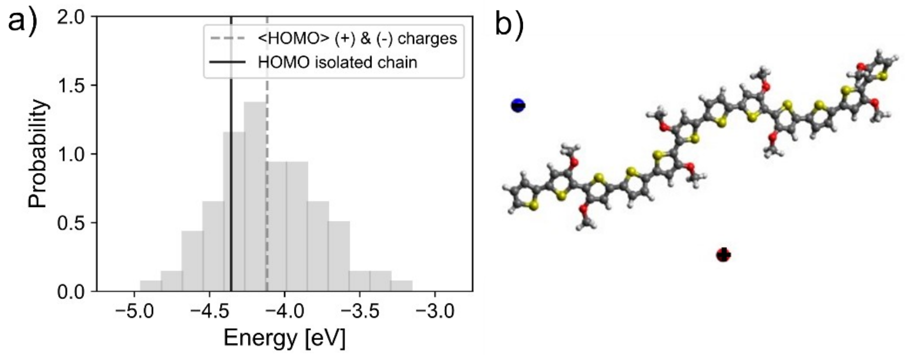

Figure S6: (a) Distribution of chain HOMO energy for 100 samples of a model chain surrounded by two equal and opposite point charges compared with the HOMO energy of the isolated chain. (b) Example of a structure containing the central chain and two surrounding point charges.

S7 Time evolution of charge in a dynamic disordered landscape

This section describes the steps required to perform the simulation of the dynamics of a single carrier in a disordered landscape where the on-site energies are fluctuating in time.

S7.1 Numerical integration of 1st order rate equation with time dependent rates

The differential rate equation is:

$$\frac{dP_j}{dt} = -\sum_{i \neq j} k_{j \to i} P_j + \sum_{i \neq j} k_{i \to j} P_i$$

Where P_j is the population in state j. We will be using this expression to describe charge hopping so a different state *j* corresponds to a different location in space.

Suppose that rates are time dependent i.e $k_{j \to i} \equiv k_{j \to i}(t)$. The differential can be replaced with a finite difference $\frac{dP_j}{dt} \simeq \frac{P_j(t_{n+1}) - P_j(t_n)}{t_{n+1} - t_n}$ and time discretised so that $t_n = n\delta t$ (and therefore $t_{n+1} - t_n = \delta t$). The first equation becomes

$$P_j(t_{n+1}) = P_j(t_n)\left(1 - \sum_{i \neq j} k_{j \to i}(t_n)\delta t\right) + \sum_{i \neq j} k_{i \to j}(t_n)\delta t P_i(t_n)$$

and the numerical procedure is accurate as long as δt is chosen to be small enough that $\sum_{i \neq j} k_{j \to i}(t_n)\delta t \ll 1$ (otherwise one can get negative populations).

S7.2 Rates in the presence of time dependent on-site energy

In this work we resort to the Miller-Abrahams rate expression shown below:

$$k_{j \to i}(t) = \begin{cases} k_0(R_{ij}) & if \quad E_i(t) \leq E_j(t) \\ k_0(R_{ij})\exp\left((E_j(t) - E_i(t))/k_B T\right) & otherwise \end{cases}$$

Where $k_0(R_{ij})$ depends on the distance between states *i* and *j*.

S7.3 Generating a sequence of normally distributed values with various degrees of correlation

Consider a particle in 1D with coordinate *x*, experiencing a potential energy $V(x) = \frac{1}{2}ax^2$ and moving in the overdamped regime (i.e. large friction). The trajectory of this particle can be described by the following algorithm. At time t_n the particle is in position $x(t_n)$ and has energy $V_{initial} = \frac{1}{2}ax(t_n)^2$. A small displacement Δx (can be positive or negative) is generated randomly and the corresponding new energy is computed as $V_{final} = \frac{1}{2}a(x(t_n) + \Delta x)^2$. The new position is accepted with probability $\min\left(1, \exp\left((V_{initial} - V_{final})/k_B T\right)\right)$. If accepted, the new position will be $x(t_{n+1}) = x(t_n) + \Delta x$ otherwise the new position will be $x(t_{n+1}) = x(t_n)$. This algorithm produces a sequence of $x(t_n)$ distributed normally with standard deviation $\sqrt{k_B T/a}$. It may be convenient to set $k_B T = a = 1$ and consider the algorithm in this paragraph as a means to generate a sequence of $x(t_n)$ distributed normally with unit standard deviation. The parameter Δx determines how correlated the sequence is ($\Delta x > \sqrt{k_B T/a}$ produces an uncorrelated sequence and $\Delta x << \sqrt{k_B T/a}$ produces a very correlated sequence).

If one wants to generate a sequence of energies with standard deviation σ_E they are obtained from the sequence of $E(t_n) = \sigma_E x(t_n)$. As there are many energies that fluctuate, we use the notation $E_j(t_n) = \sigma_E x_j(t_n)$. The rate in the general case can be written as

$k_{j\to i}(t) = f(k_0(R_{ij}), \sigma_E, x_i(t), x_j(t))$

S7.4 Units for the implementation and parameters range

Considering a lattice of sites with distance d, $k_0(d)$ is the fastest hopping rate and it is set to 1, i.e. $1/k_0(d)$ is the unit of time. $k_B T = 1$, i.e. it is the unit of energy.

δt is the integration step, which should be chosen so that the results do not depend on its value. It this work it was chosen to be 0.05, i.e. 20 times shorter than the fastest hopping time.

σ_E is the standard deviation of the energy, it will be much larger than $k_B T$ and a starting value could be 15 $k_B T$. In these units $E_j(t) - E_i(t) = \sigma_E(x_j(t) - x_i(t))$

Δx determines how rapidly the energy changes. The implementation is much easier if the energy is updated every integration step δt. The parameter is best considered as $\Delta x/\delta t$ because $1/\delta t$ is the number of integration steps that take place in one typical hopping time and $\Delta x/\delta t$ is therefore a measure of how much the energy moves in a typical hopping time. $\Delta x/\delta t \ll 1$ represents energy moving more slowly than the hopping time while $\Delta x/\delta t \sim > 1$ is energy moving as rapidly as the hopping time. In the main manuscript, we reported as a parameter of the model τ_{fluct} rather than Δx, defined as the time required for the autocorrelation function of the on-site energy to become 0.5 (i.e. a characteristic time for the fluctuation for easier interpretation of the results).

S7.5 Initialization of on-site energy

It is convenient that at the beginning of the simulation the particle is in a site with energy drawn from the correct distribution $P(x) \sim \exp(-x^2/2)\exp(-x\sigma_E) = \exp((-x^2 - 2x\sigma_E)/2)$, which is a Gaussian distribution of standard deviation 1 centered on $x = -\sigma_E$. In the code this means that the site where the carrier is placed is decreased in energy by $-\sigma_E$.

References

(1) Landi, A.; Reisjalali, M.; Elliott, J. D.; Matta, M.; Carbone, P.; Troisi, A. Simulation of Polymeric Mixed Ionic and Electronic Conductors with a Combined Classical and Quantum Mechanical Model. *J. Mater. Chem. C* **2023**, *11* (24), 8062–8073. https://doi.org/10.1039/D2TC05103F.
